# Enhancing soybean germination and vigor under water stress: the efficacy of bio-priming with sodium carboxymethyl cellulose and gum arabic

**DOI:** 10.3389/fpls.2024.1475148

**Published:** 2025-01-03

**Authors:** Aisha Almakas, Ahmed S. Elrys, El-Sayed M. Desoky, Laila A. Al-Shuraym, Sadeq K. Alhag, Mohammed O. Alshaharni, Fawze Alnadari, Zhang NanNan, Zunaira Farooq, Khaled A. El-Tarabily, Tuanjie Zhao

**Affiliations:** ^1^ National Center for Soybean Improvement, Key Laboratory of Biology and Genetics and Breeding for Soybean, Ministry of Agriculture, State Key Laboratory for Crop Genetics and Germplasm Enhancement, Nanjing Agricultural University, Nanjing, China; ^2^ Research and Development Center, Jiangsu Tianmeijian Nature Bioengineering Co., Ltd., Nanjing, China; ^3^ Soil Science Department, Faculty of Agriculture, Zagazig University, Zagazig, Egypt; ^4^ School of Tropical Agriculture and Forestry, Hainan University, Haikou, China; ^5^ Botany Department, Faculty of Agriculture, Zagazig University, Zagazig, Egypt; ^6^ Biology Department, Faculty of Science, Princess Nourah bint Abdulrahman University, Riyadh, Saudi Arabia; ^7^ Biology Department, College of Science and Arts, King Khalid University, Muhayl Asser, Saudi Arabia; ^8^ Biology Department, College of Science, King Khalid University, Abha, Saudi Arabia; ^9^ Department of Biology, College of Science, United Arab Emirates University, Al Ain, United Arab Emirates

**Keywords:** biopolymers, drought stress, flooding stress, osmotic adjustment, seed priming, water scarcity

## Abstract

Seed priming can significantly enhance the tolerance of soybean against different environmental stresses by improving seed water uptake and modulating stress-response mechanisms. In particular, seed priming with sodium carboxymethylcellulose (SCMC) and gum Arabic (GA) can support seeds to withstand extreme conditions better, promoting more consistent germination and robust seedling establishment, which is crucial for achieving stable agricultural yields. The present study investigated the effects of seed priming using a combination of SCMC and GA (10% CG) on the germination, growth, and biochemical responses of six soybean varieties under drought and flooding stress conditions. The results revealed significant differences among varieties and applied treatments on germination, vigor, and physiological traits. Under drought stress, seed priming with 10% CG significantly improved germination percentage, germination rate, shoot length, root length, and biomass compared to unprimed seeds. Notable reductions in malondialdehyde (MDA) content and enhanced antioxidant enzyme activities, including superoxide dismutase (SOD), catalase (CAT), and peroxidase (POD), suggest that 10% CG priming mitigates oxidative damage through enhanced antioxidant defense mechanisms. Moreover, 10% CG seed priming improved germination and growth parameters under flooding stress, but the advantages were less significant. In addition, the priming treatment significantly reduced electrolyte conductivity (EC) across all varieties compared to unprimed seeds, indicating improved membrane stability. Overall, 10% CG seed priming was more effective under drought and flooding conditions, demonstrating a potential strategy for enhancing stress tolerance in soybean varieties.

## 1 Introduction

Soybean (*Glycine max*) is essential for global nutrition and agriculture, serving as a major source of protein, essential fatty acids, and various nutrients (Toomer et al., 2023). It plays a critical role in human diets and is a fundamental component of animal feed, contributing significantly to food security and agricultural sustainability worldwide (Anderson et al., 2019). Additionally, its ability to fix nitrogen naturally enriches soil fertility, making it beneficial for sustainable agricultural practices (Dinar et al., 2019; Korobko et al., 2024). However, drought stress negatively impacts soybean germination by reducing seed water uptake, leading to delayed or incomplete germination and poor seedling establishment (Kakati et al., 2022). Besides, during early developmental stages, insufficient water availability impedes root and shoot growth, weakening the plants and reducing their ability to absorb essential nutrients (Xiong et al., 2021). In addition, at the reproductive stage, drought stress disrupts flowering and pod formation, resulting in decreased seed set, lower seed quality, and significantly reduced yield (Yerzhebayeva et al., 2024).

The primary mechanisms through which drought stress affects plants include reduced water availability, which disrupts CO_2_ fixation and impairs photosynthetic efficiency (Qiao et al., 2024). This occurs as a result of cellular water loss, causing the closure of leaf stomata to conserve moisture, which in turn reduces gas exchange and slows plant growth (Desoky et al., 2023; Qiao et al., 2024). Additionally, drought stress leads to the overproduction of reactive oxygen species (ROS) like O_2_^•−^ and H_2_O_2_, which induce oxidative damage to DNA, lipids, and proteins, further inhibiting photosynthesis and slowing development (Alharby et al., 2021; Abd El-Mageed et al., 2022). Disruptions in water relations, coupled with oxidative stress, impact plant metabolism, physiological and biochemical processes, and hormonal balance, necessitating methods to improve drought resilience (Alharby et al., 2021; Rady et al., 2021).

Furthermore, flooding stress poses severe challenges to soybean growth but through different mechanisms (Yijun et al., 2022). Excess water during flooding or submergence results in oxygen deprivation in the root zone, impeding respiration and energy production critical for root and shoot development (Habibullah et al., 2021; Nasrullah et al., 2022). This lack of oxygen disrupts normal cellular metabolism and reduces plant ability to uptake nutrients from the soil, causing nutrient imbalances and stunted growth (Rupngam and Messiga, 2024). Moreover, flooding often leads to the accumulation of ethylene, a stress hormone that accelerates leaf senescence and can cause premature tissue death (Manghwar et al., 2024). Plants under flooding stress must also cope with altered carbohydrate metabolism and impaired signalling pathways, which compromise the energy reserves needed to maintain growth and physiological functions (Yang et al., 2023).

Seed priming is an effective strategy for enhancing seed germination and seedling vigor, especially under stressful environmental conditions such as water stress and flooding (El-Sanatawy et al., 2021a). In this context, natural polymers like sodium carboxymethyl cellulose (SCMC) and gum Arabic (GA) gained attention for their role in mitigating the adverse effects of these stresses (Saberi Riseh et al., 2023). SCMC, a water-soluble cellulose derivative, and GA, a biopolymer obtained from *Acacia* trees, are known for their biocompatibility and role in modulating stress responses (Badwaik et al., 2022). Priming using these agents has the potential to improve the physiological and biochemical responses of the seeds, thereby enhancing germination rates (GR) and seedling vigor (Chin et al., 2021; Paul and Rakshit, 2023).

Under water stress, these natural polymers create a favorable microenvironment that enhances water uptake, stabilizes cellular membranes, and activates antioxidant defences, leading to improved seedling establishment (Azeem et al., 2023). Conversely, under flooding conditions, SCMC and GA facilitate better oxygen availability and enhance metabolic activities, which are crucial for maintaining seed viability and growth (Dingley et al., 2024). SCMC and GA have promising impacts in fortifying plant defence mechanisms, particularly under adverse conditions. These agents act by enhancing the activity of key antioxidant enzymes, including catalase (CAT), superoxide dismutase (SOD), and peroxidase (POD) (Shakir et al., 2022). Antioxidant enzymes like CAT, SOD, and POD play a crucial role in neutralizing ROS generated during stress, thereby preventing cellular damage and promoting plant resilience (Hasanuzzaman et al., 2020; Desoky et al., 2023; Mansour et al., 2023).

The primary objective of the current study was to investigate the effects of priming soybean seeds with SCMC and GA on germination and early growth under water stress and flooding conditions. The study involved a comprehensive analysis of GR, seedling development, and physiological responses of primed seeds using a combination of SCMC and GA (10% CG) to assess the role of these biopolymers in enhancing seed resilience to drought and flooding stressors. Additionally, the work also explored the underlying mechanisms associated with antioxidant defense and dehydration response during seed germination under adverse conditions. By elucidating these processes, this study aimed to demonstrate the potential of biopolymer priming as a strategy to mitigate the negative effects of water-related stress on soybean cultivation.

## 2 Materials and methods

### 2.1 Plant material

Six different soybean varieties were used to optimize nutrient seed priming in soybean underlying seed drought tolerance (**Table 1**). The evaluated varieties have different growth periods, seed size, and other morphological traits. All the soybean varieties were obtained from Chinese National Center for Soybean Improvement (CNCSI), Nanjing Agricultural University, Jiangsu, China. Seed priming used two biopolymers SCMC (CAS: 9004–32-4, viscosity: 300–800 mPa·s) was purchased from Sino Pharm Chemical Reagent Co., Ltd. (Shanghai, China). GA, molecular weight 250 kDa was obtained from the Shanghai Ryon Biological Technology Co., Ltd. (Shanghai, China). All other reagents used were of analytical grade.

**Table 1:** Characteristics of soybean varieties used in the current study.

Code	Name	Planting type	Seed coat color	100-seed weight
T21R1001	Tianlong Number 1	Spring	Yellow	19.05 g
T21R1003	NJ71-1	Spring	Yellow	15.40 g
T21R1009	You 1511	Spring	Yellow	18.14 g
T21R1006	NJ26-3	Spring	Yellow	16.32 g
T21R1012	NJ17-33	Summer	Black	21.46 g
T21R1015	NJ59-1	Summer	Black	17.53 g

Source: All soybean varieties were obtained from the Chinese National Center for Soybean Improvement (CNCSI), Nanjing Agricultural University, Jiangsu, China.

### 2.2 Characterization of biopolymers

#### 2.2.1 Scanning electron microscopy analysis

The CG samples were mounted on a bronze stub and sputtered with a thin layer of gold. The surface section morphology of the samples was observed by using Philips XL-30 scanning electron microscopy (SEM; FEI Co., Eindhoven, The Netherlands) with an acceleration voltage of 10 kV.

#### 2.2.2 Fourier transform-infrared (FT-IR) spectroscopy analysis

FT-IR spectrometer (Nicolet iS-50, Thermo Fisher Scientific, MA, USA) was used to characterize the presence of functional groups in the powder of SCMC, GA and CG samples. The spectra were collected in a wavelength range of 4000–525 cm^−1^ by averaging 32 scans at a resolution of 4 cm^−1^.

#### 2.2.3 X-ray diffraction

An X-ray diffractometer (D8 Advance, Bruker, USA) was used to identify the crystal structure of the CG samples at 30 kV and 10 mA. Angular range (2θ = 5 – 80 degrees) was applied to detect the scattered radiation at 15.6 degrees min^−1^ scanning speed.

#### 2.2.4 Differential scanning calorimetry analysis

DSC (DSC-60, Shimadzu Corp., Kyoto, Japan) was used to analyze the thermal properties of the CG samples. Briefly, 10 mg of CG was sealed in a standard aluminum pan which was heated under a nitrogen atmosphere from 27 to 450°C at a rate of 10°C min^−1^.

### 2.3 Seed priming and preparation

In a preliminary experiment, various concentrations were tested to determine the optimal concentration for enhancing seed performance. Through systematic evaluation, 10% CG was identified as the most effective concentration. Soybean seeds were surface sterilized using 20% Clorox bleach, and 70% ethyl alcohol (Sigma-Aldrich Chemie GmbH, Taufkirchen, Germany). These seeds were then washed ten times with 0.2 µm Millipore membrane (Millipore Corporation, MA, USA) filter-sterilized distilled water.

Soybean seeds were soaked in 10% CG for 6 h. After priming, the seeds were left to dry before germination, four interventions were included in the experiment, each replicated three times in a completely randomized design (CRD).

### 2.4 Germination under drought stress

Seed germination was applied using the paper roll method employing a solution of polyethylene glycol (PEG, Sigma-Aldrich) to induce drought stress during a seven-day cultivation period. Fifteen seeds of each variety were wrapped in Whatman filter paper (Whatman, Maidstone, England) and immersed in a 20% (w/v) solution of PEG 6000 to simulate drought conditions. For the control treatment, both primed and non-primed seeds were grown under normal conditions without any imposed stress. Each treatment was replicated three times to ensure reliability.

The paper rolls were randomly arranged in a growth chamber set to maintain an average day/night temperature of 22/19°C and 75% relative humidity for seven days (Vassilevska-Ivanova et al., 2014; Salleh et al., 2020). All soybean varieties were phenotypically evaluated for various traits including germination percentage (GP), GR, shoot length (ShL), root length (RL), seedling fresh weight (SFW), and seedling dry weight (SDW). The GP and GR of the samples at three and seven days were calculated as follows:


$$\text{GP or GR }\left(\%\right)=\left(\frac{\text{Number of seeds germinated ​}}{\text{Total number of seeds}}\right)\;\times 100\;$$

### 2.5 Germination under flooding stress

High-quality seeds were surface sterilized as described above. The experiment was organized into four treatment groups in CRD. Twenty seeds from each variety were placed in 350 mL plastic cups containing 50 mL of distilled water, covered with sterilized Petri dishes, and incubated for three days in a germination cabinet maintained at 25°C, following the protocol of Ali et al. (2018).

During this incubation period, electrical conductivity (EC) was measured daily to assess membrane integrity by monitoring ion leakage, such as potassium and sodium, which serves as an indicator of membrane damage and cell permeability. After three days, seeds demonstrating intact membranes, indicative of high quality and viability, were selected and transferred onto germination paper. The paper was then rolled to enclose the seeds, and the rolls were maintained for an additional seven days. Subsequently, various parameters were measured, including GR, ShL, RL, SFW, and SDW under both control and seed flooding conditions.

### 2.6 Measurements of antioxidant enzyme activities

The activities of POD, SOD, and CAT, along with the malondialdehyde (MDA) content, were measured using a POD assay kit (A084-3), SOD assay kit (T-SOD, A001-1), CAT assay kit (A007-1), and MDA assay kit (A003), respectively.

All assays were conducted according to the manufacturer instructions provided by the Nanjing Jiancheng Bioengineering Institute (Nanjing, China), with each biological replicate performed in triplicate. In brief, 1.0 g of fresh root tissue was ground into a fine paste using a mortar and pestle. The paste was then mixed with 9 mL of ice-cold 20× phosphate-buffered saline (PBS) solution (Beijing Solarbio Science & Technology Co., Ltd., Beijing, China) with a pH of 7.2–7.4. The resulting homogenates were centrifuged at 3500 rpm for 10 min at 4°C. The supernatants were collected and used as crude extracts for the assays, with measurements conducted using a UV-1800 (Shimadzu Corporation, Analytical Instruments Division, Kyoto, Japan).

### 2.7 Statistical analysis

All experiments were replicated thrice and expressed as mean ± SD. Experimental data were statistically analyzed using SPSS 16.0 software (SPSS Inc., IBM SPSS Statistics, Chicago, IL, USA). Analysis of a two-factor completely randomized was used to evaluate the significance. Data were analyzed using Duncan’s multiple range test at 1% level of significance.

## 3 Results

### 3.1 Characterization of biopolymer properties

**Figure 1:**
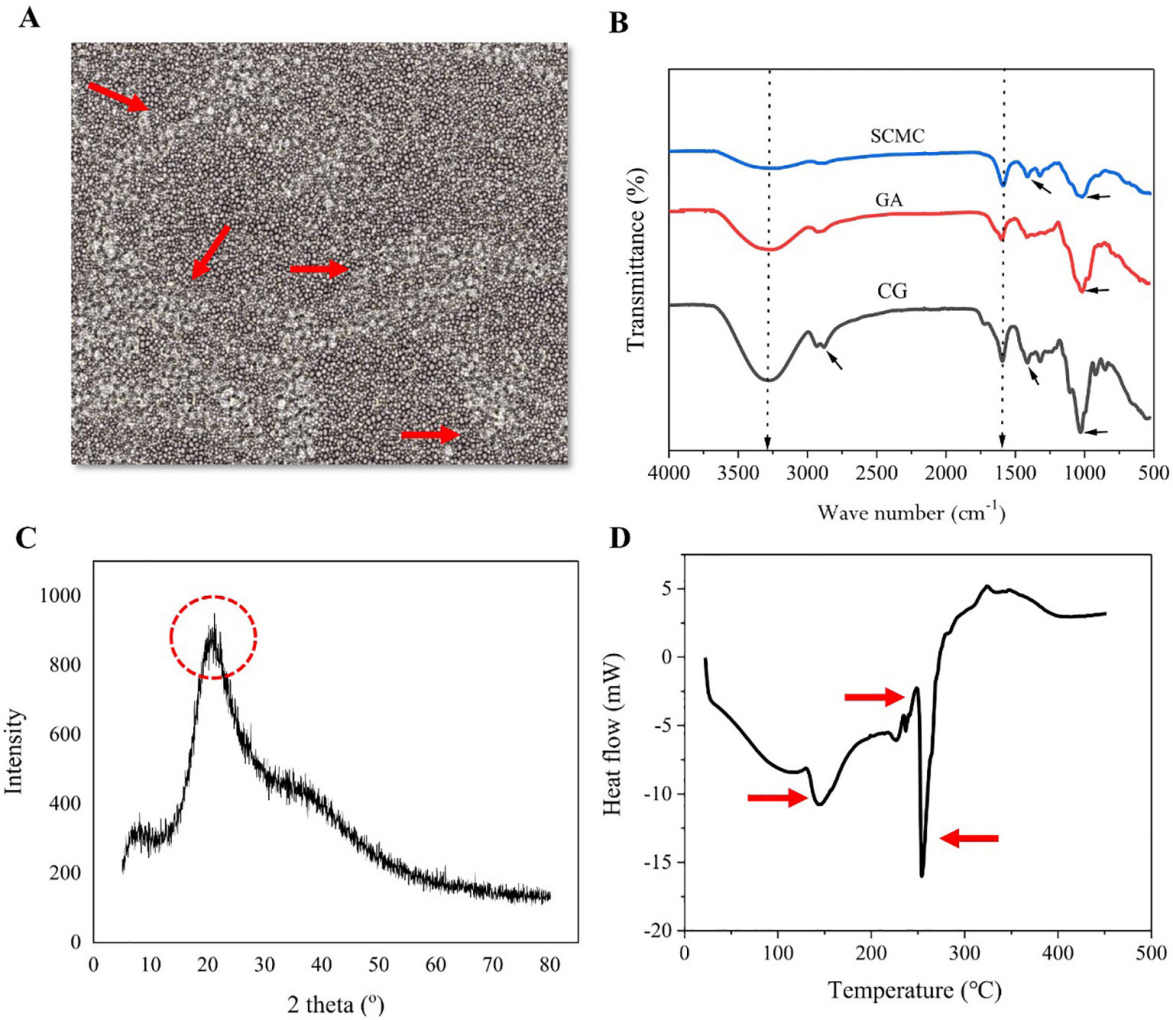
Surface-sectioned scanning electron microscopy (SEM) showing the surface section and cross-section morphologies of biopolymers **(A)**. Fourier transform-infrared (FTIR) spectra of SCMC, GA, and 10% CG **(B)**, X-ray diffraction (XRD) pattern of CG biopolymers **(C)**, and differential scanning calorimetry (DSC) thermograms of CG biopolymers **(D)**. SCMC, sodium carboxymethyl cellulose; GA, gum Arabic; CG, SCMC incorporated with GA.

**Figure 1A** depicts the microstructure of the biopolymer composed of SCMC and GA (CG). The arrows indicate areas that exhibit essential surface attributes, including texture uniformity and particle dispersion, which are vital for evaluating material homogeneity and smoothness. The FT-IR spectra of SCMC and GA biocomposite biopolymer and SCMC biocomposite containing 10% GA are depicted in **Figure 1B**.

Broadband emission near 1590 cm^−1^ was attributed to the N–H bending resonance in the case of SCMC, which overlapped the amide II vibration. The bands detected at 1419 and 1053 cm^−1^ were caused by the stretching vibration of the C–O–C bond in the glycosidic linkage of the polymer and the scissoring vibration of the NH_2_ group, respectively (**Figure 1B**). The spectra of the GA and the CG biocomposite biopolymer were nearly identical due to the structural similarity of the polysaccharides. In contrast, the introduction of GA into SCMC resulted in a modification of the wavelength values when compared to GA (3287 cm^−1^), thereby establishing a robust absorption band at 3276 cm^−1^ that facilitated O–H stretching (**Figure 1B**).

The expansive bond observed at 3276 cm^−1^ was determined to result from elongation and unconstrained hydroxyl group vibrations within and between molecules. Furthermore, the presence of an additional peak (2878 cm^−1^) in the spectrum, which was absent in both the SCMC and GA spectra, suggested the possibility of a cross-linked reaction between the two substances. The peak shifted marginally from 3277 to 3282 cm^−1^ following the addition of GA, indicating that the surface OH of GA and the OH of CG molecules were in interaction. Collectively, these findings indicated that the incorporation of GA compounds into the SCMC was useful for enhancing the mechanical and barrier properties of the biocomposite.

In addition, XRD analysis shows the successful incorporation of GA into the SCMC biopolymer (**Figure 1C**). The region in question exhibits no discernible peaks besides the peak at a diffraction angle of 20 degrees resulting in a decrease in crystallinity. In addition, the results of the DSC analysis showed an improvement in the thermal stability of the biopolymer after adding GA, which could decrease the quantity of hydroxyl groups present in polysaccharides (**Figure 1D**). This causes the biopolymer’s structure to be enhanced.

### 3.2 Effect of seed priming on soybean germination under drought and flooding stresses

The analysis of variance (ANOVA) results presented in **Table 2** revealed significant effects of assessed varieties and treatments on the studied traits of soybean under drought and flooding stress conditions. Under drought stress, highly significant differences (*P*< 0.01) were observed among varieties and treatments for all parameters, including GR, GP, ShL, RL, SFW, SDW, MDA content, CAT activity, SOD activity, and POD activity (**Table 2**). The interaction between varieties and treatments was also significant for most traits, such as ShL, RL, SFW, SDW, MDA content, CAT activity, SOD activity, and POD activity, indicating that the response to drought stress varied across the different soybean varieties (**Table 2**).

**Table 2:** Mean squares of studied traits for treated soybean varieties with sodium carboxymethyl cellulose and gum Arabic (10% CG) under drought and flooding conditions.

Source of variance	Varieties (V)	Treatment (T)	V×T	Error	Total
Degree of freedom	5	3	15	48	71

Under drought stress				
Germination percentage	911.63 ^**^	2061.55 ^**^	10.67 ^NS^	11.74	161.49
Germination rate	2148.32 ^**^	3073.18 ^**^	34.26 ^*^	14.2	297.98
Shoot length	71.69 ^**^	64.48 ^**^	1.59 ^**^	0.27	8.29
Root length	114.78 ^**^	230.21 ^**^	3.42 ^**^	0.54	18.9
Seedling fresh weight	0.19 ^**^	0.21 ^**^	0 ^**^	0	0.02
Seedling dry weight	0.00093 ^**^	0.00077 ^**^	0.00004 ^**^	2.6E-06	0.00011
Malondialdehyde content	122.53 ^**^	1473.4 ^**^	29.71 ^**^	0.65	77.61
Catalase activity	1284.09 ^**^	2357.46 ^**^	185.27 ^**^	1.34	230.08
Superoxide dismutase activity	5663.6 ^**^	36799.6 ^**^	2522.1 ^**^	139.81	2581.08
Peroxidase activity	711.39 ^**^	2099.52 ^**^	84.1 ^**^	1.08	157.31
	Under flooding stress				
Germination rate	1006.5 ^**^	1467.31 ^**^	13.94 ^NS^	9.25	142.0783
Shoot length	30.4069 ^**^	46.7967 ^**^	0.3778 ^**^	0.0233	4.214239
Root length	76.165 ^**^	171.93 ^**^	1.099 ^**^	0.014	12.8702
Seedling fresh weight	0.10958 ^**^	0.23418 ^**^	0.00236 ^**^	0.00004	0.018139
Seedling dry weight	0.000259 ^**^	0.0015 ^**^	6.97E-06 ^**^	1.72E-06	8.42E-05

NS, Not significant, *P< 0.05, **P< 0.01.

Under flooding stress, highly significant differences differences (*P*< 0.01) were also observed for GR, GP, ShL, RL, SFW, and SDW among varieties and treatments (**Table 2**). The interaction between soybean varieties and treatments was significant for ShL, RL, SFW, and SDW, suggesting variability in the response of different soybean varieties to flooding stress (**Table 2**). Seed priming with 10% CG under drought stress treatment demonstrated a significant improvement in the germination parameters of assessed soybean varieties compared to the untreated control. Under normal conditions without any induced drought stress, seeds pretreated with 10% CG exhibited higher GR and GP than the untreated seeds, highlighting the effectiveness of the seed priming strategy (**Table 2**). This indicates that 10% CG priming enhanced the seeds water uptake and metabolic activation, leading to more efficient germination.

**Figure 2:**
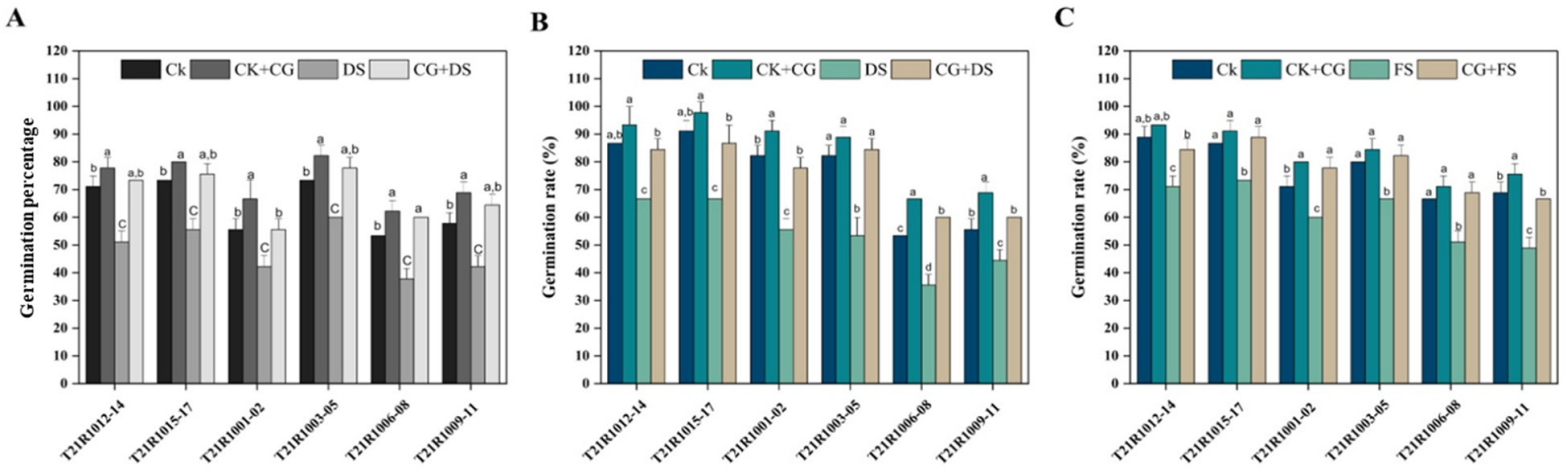
Effects of seed priming using a combination of sodium carboxymethyl cellulose and gum Arabic (10% CG) on soybean germination under drought and flooding stresses. **(A)** germination percentage (GP) of assessed six soybean varieties under drought stress. **(B)** germination rate (GR) of assessed six soybean varieties under drought stress conditions. **(C)** germination rate (GR) of assessed six soybean varieties under flooding stress. Vertical bars above columns indicate the standard deviation (SD). Mean values followed by different letters are significantly (*P*< 0.01) different from each other according to Duncan’s multiple range test. CK, control seeds (seeds pretreated with H_2_O germinated under normal conditions without any imposed stress); CK+CG, seeds pretreated with 10% CG germinated under normal conditions; DS, seeds pretreated with H_2_O germinated under drought stress; FS, seeds pretreated with H_2_O germinated under flooding stress; CG+DS, seeds pretreated with 10% CG under drought stress; CG+FS, seeds pretreated with 10% CG under flooding stress.

Under drought stress, seeds pretreated with 10% CG maintained relatively higher GR and GP compared to the control (**Figures 2A, B**). This suggests that seed priming provides a protective mechanism, possibly through osmotic adjustment or the activation of stress-related pathways, that enables seeds to better withstand drought conditions.

In contrast, the physiological response to flooding stress revealed a different pattern. Under normal conditions, 10% CG seed priming still enhanced GR as observed in the control (**Figure 2C**). However, when exposed to flooding stress, the GP of seeds pretreated with 10% CG was slightly lower than the control group. This indicates that while seed priming generally boosts germination under optimal conditions, its efficacy diminishes under flooding stress.

Flooding can reduce seed respiration and oxygen availability, which 10% CG priming cannot fully address. The essential contention is that although seed priming has certain metabolic advantages, its effects are less significant under flooding stress than under drought conditions. This suggests that flooding presents a more complex challenge requiring additional or alternative strategies, such as the development of varieties with enhanced anaerobic germination tolerance or improved field management practices to prevent waterlogging.

### 3.3 Effects of seed priming on seedlings traits under drought and flooding stresses

**Figure 3:**
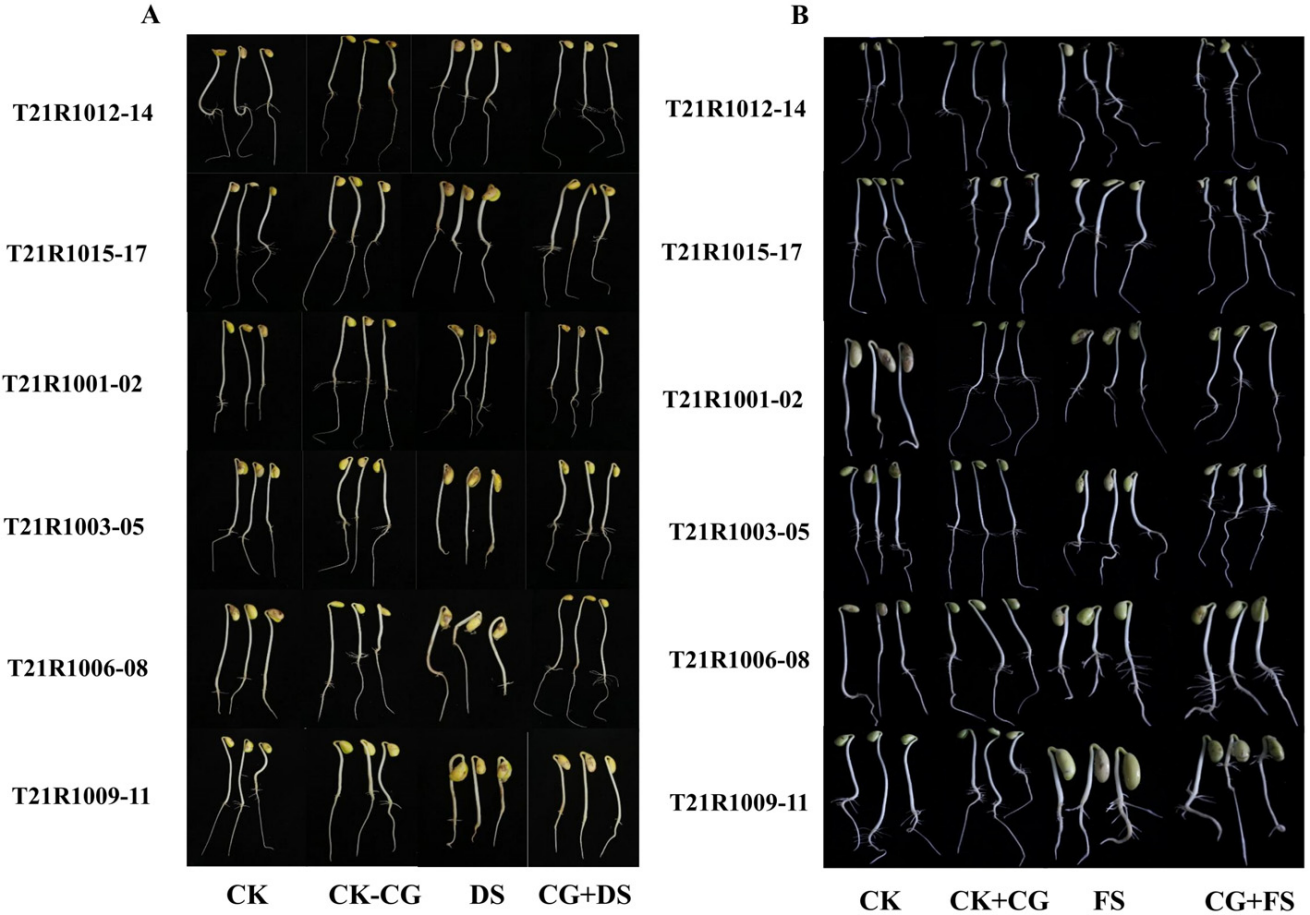
Effects of seed priming using a combination of sodium carboxymethyl cellulose and gum Arabic (10% CG) on seedling traits of assessed six soybean varities under drought **(A)** and flooding **(B)** stresses. CK, control seeds (seeds pretreated with H_2_O germinated under normal conditions without any imposed stress); CK+CG, seeds pretreated with 10% CG germinated under normal conditions; DS, seeds pretreated with H_2_O germinated under drought stress; FS, seeds pretreated with H_2_O germinated under flooding stress; CG+DS, seeds pretreated with 10% CG under drought stress; CG+FS, seeds pretreated with 10% CG under flooding stress.

Primed soybean seedlings demonstrated minimal phenotypic changes, maintaining fully expanded seedling structures across all priming treatments compared to the control (without seed stress) in six distinct soybean varieties (**Figures 3A, B**). These results suggest that seed priming effectively mitigates the adverse effects of water stress on the morphological traits of soybean seedlings. The ability to sustain fully expanded seedlings indicates that the priming treatments supported overall growth and development even under challenging drought and flooding conditions (**Figures 3A, B**).

**Figure 4:**
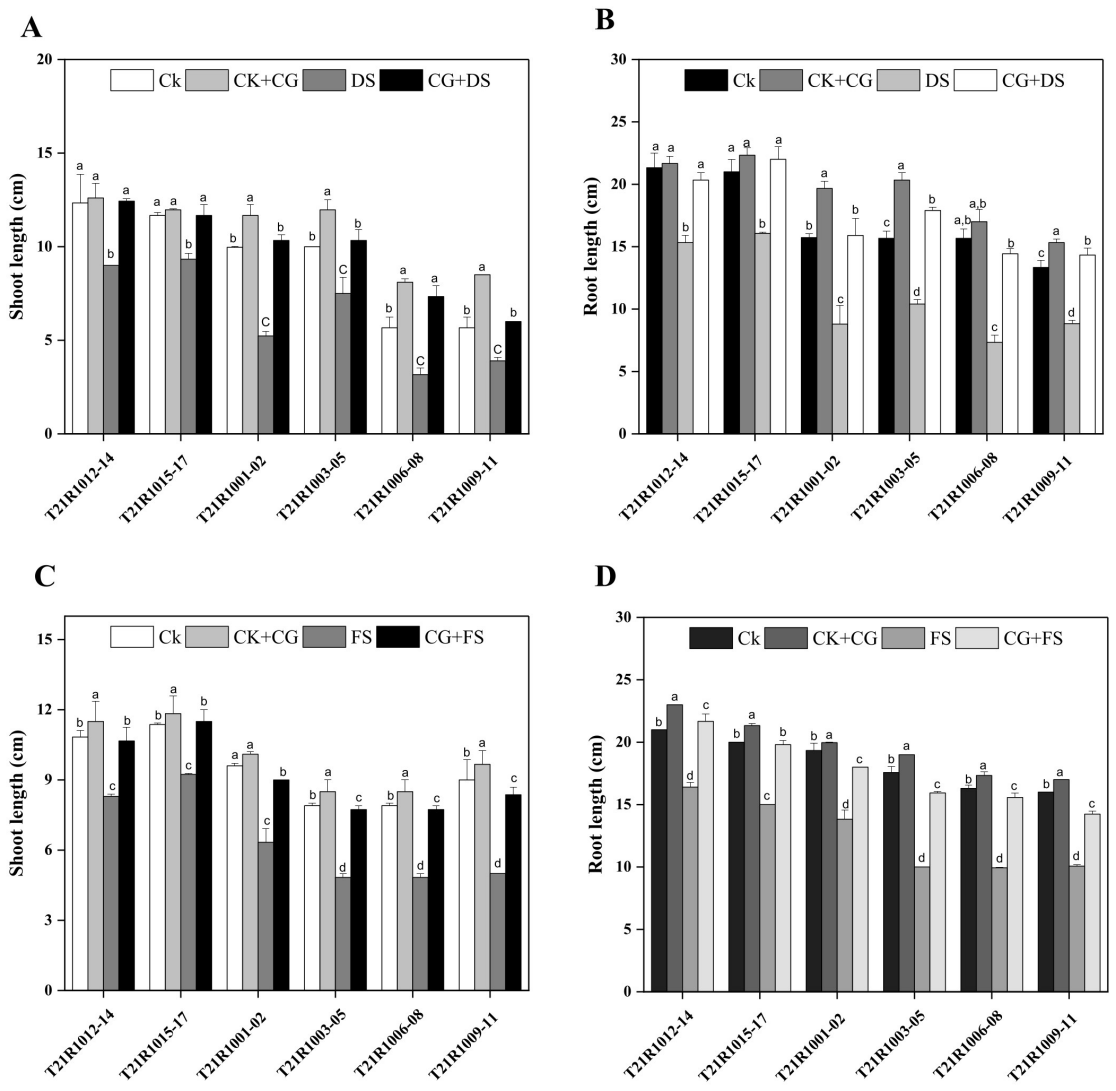
Impacts of seed priming using a combination of sodium carboxymethyl cellulose and gum Arabic (10% CG) on shoot length under drought stress **(A)**, root length under drought stress **(B)**, shoot length under flooding stress **(C)** and root length under flooding stress **(D)** of assessed six soybean varieties. Vertical bars above columns indicate the standard deviation (SD). Mean values followed by different letters are significantly (*P*< 0.01) different from each other according to Duncan’s multiple range test. CK, control seeds (seeds pretreated with H_2_O germinated under normal conditions without any imposed stress); CK+CG, seeds pretreated with 10% CG germinated under normal conditions; DS, seeds pretreated with H_2_O germinated under drought stress; FS, seeds pretreated with H_2_O germinated under flooding stress; CG+DS, seeds pretreated with 10% CG under drought stress; CG+FS, seeds pretreated with 10% CG under flooding stress.

Measurements of ShL and RL for different soybean varieties without seed priming showed a substantial decline after seven days of drought and flooding stress (**Figure 4**). Statistical analysis revealed significant differences among the control group (CK) under normal conditions, CK+CG under normal conditions, and treatments under drought and flooding stress, as well as CG and drought stress under primed stress conditions. The results demonstrated that ShL and RL were significantly enhanced by all seed priming treatments under drought and flooding stress (**Figure 4**).

Notably, ShL was considerably higher in all varieties with CK+CG, CG and drought stress, and CG and flooding stress seed priming treatments compared to CK, drought stress and flooding stress treatments without seed priming (**Figures 4A, C**). For RL, the T21R1001-02 variety displayed a significant increase when treated with CK+CG, CG and drought stress and CG and flooding stress, while untreated seeds (CK and S) exhibited lower RL values (**Figures 4B, D**). The findings confirmed that priming treatments significantly improved ShL and RL compared to the control (CK) and untreated drought and flooding stress treatments (**Figure 4**). Specifically, CK+CG, CG and drought stress and CG and flooding stress seed priming treatments produced notably higher ShL values (**Figure 4**).

**Figure 5:**
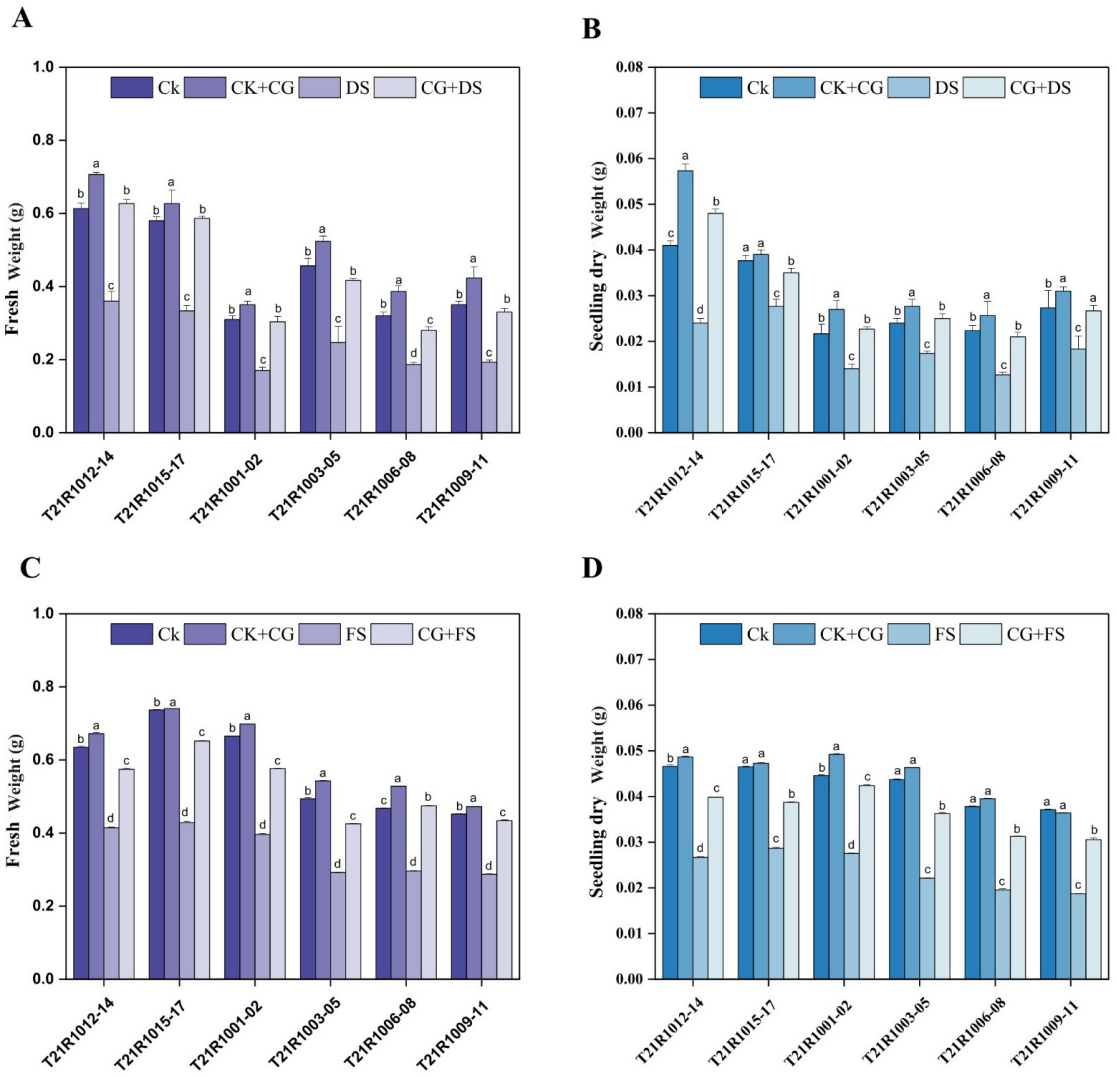
Impacts of seed priming using a combination of sodium carboxymethyl cellulose and gum Arabic (10% CG) on seedling fresh weight under drought stress **(A)**, seedling dry weight under drought stress **(B)**, seedling fresh weight under flooding stress **(C)** and seedling dry weight under flooding stress **(D)** of assessed six soybean varieties. Vertical bars above columns indicate the standard deviation (SD). Mean values followed by different letters are significantly (*P*< 0.01) different from each other according to Duncan’s multiple range test. CK, control seeds (seeds pretreated with H_2_O germinated under normal conditions without any imposed stress); CK+CG, seeds pretreated with 10% CG germinated under normal conditions; DS, seeds pretreated with H_2_O germinated under drought stress; FS, seeds pretreated with H_2_O germinated under flooding stress; CG+DS, seeds pretreated with 10% CG under drought stress; CG+FS, seeds pretreated with 10% CG under flooding stress.

Under drought stress, seeds primed with CG exhibited an increase of 16%, 54%, and 26% in both fresh and dry weight compared to unprimed seeds (**Figure 5**).

Furthermore, SFW and SDW of all soybean varieties were significantly increased by all seed priming treatments under drought and flooding stress (**Figure 5**). The highest ShL was observed in varieties treated with seed priming using SCMC and GA at a 10% concentration under water stress conditions (**Figure 4**). Overall, the data indicated that seed priming interventions, particularly those using CG, substantially enhanced ShL, root development, and increased both SFW and SDW in soybean cultivars under drought stress. Seed priming with CG at a 10% concentration was particularly effective, yielding significantly higher SFW and SDW across all varieties in drought and flooding stress conditions (**Figure 5**).

### 3.4 Effects of seed priming on oxidative damage and antioxidant defense under drought stress

**Figure 6:**
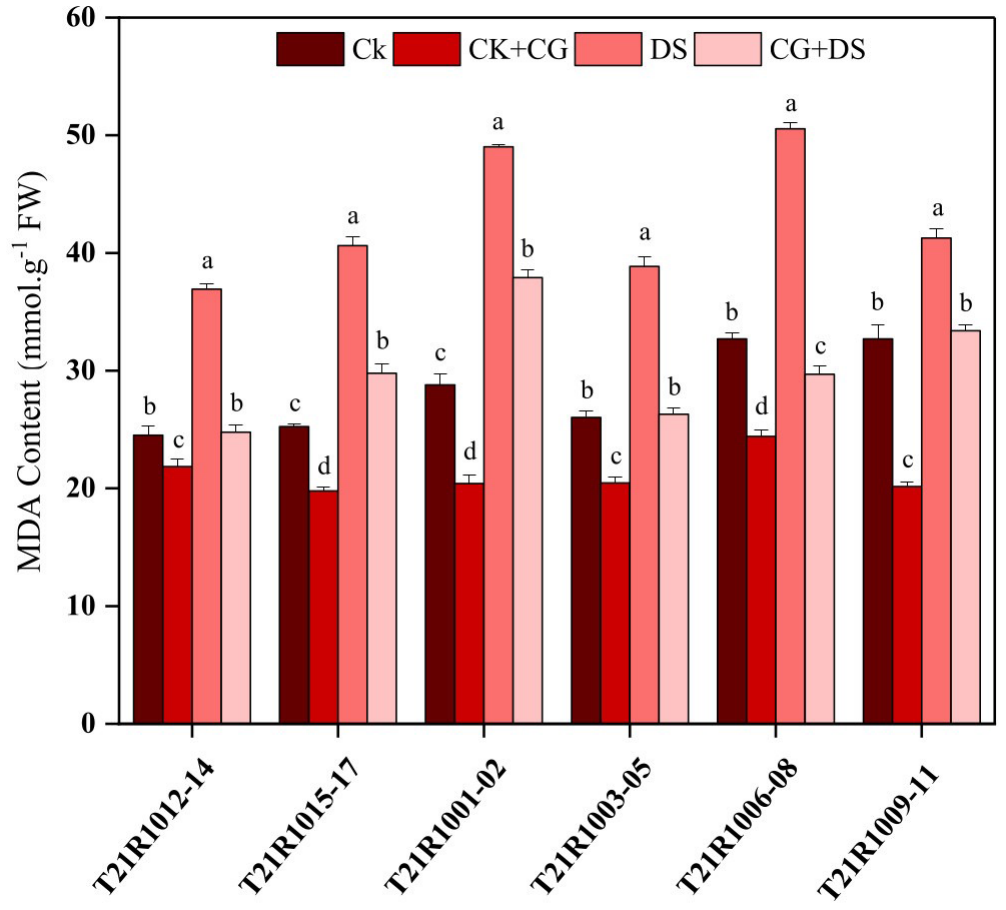
Effects of seed priming using a combination of sodium carboxymethyl cellulose and gum Arabic (10% CG) on malondialdehyde content (MDA) in soybean seedlings under drought stress. Vertical bars above columns indicate the standard deviation (SD). Mean values followed by different letters are significantly (*P*< 0.01) different from each other according to Duncan’s multiple range test. CK, control seeds (seeds pretreated with H_2_O germinated under normal conditions without any imposed stress); CK+CG, seeds pretreated with 10% CG germinated under normal conditions; DS, seeds pretreated with H_2_O germinated under drought stress; CG+DS, seeds pretreated with 10% CG under drought stress.

After seven days of germination under water-stress conditions, the seedlings exhibited a notable MDA accumulation (**Figure 6**). However, it was observed that the CK+CG treatment (seed priming with 10% CG under normal conditions without exposure to stress) followed by CG and drought stress treatment (seed priming with 10% CG under drought stress) resulted in lower MDA content compared to the drought stress treatment (seeds exposed to drought stress without CG priming) across all varieties (**Figure 6**). This finding suggests that seed priming with CG provides a protective effect against oxidative damage induced by drought stress, likely through enhanced stabilization of cellular membranes and mitigation of lipid peroxidation.

**Figure 7:**
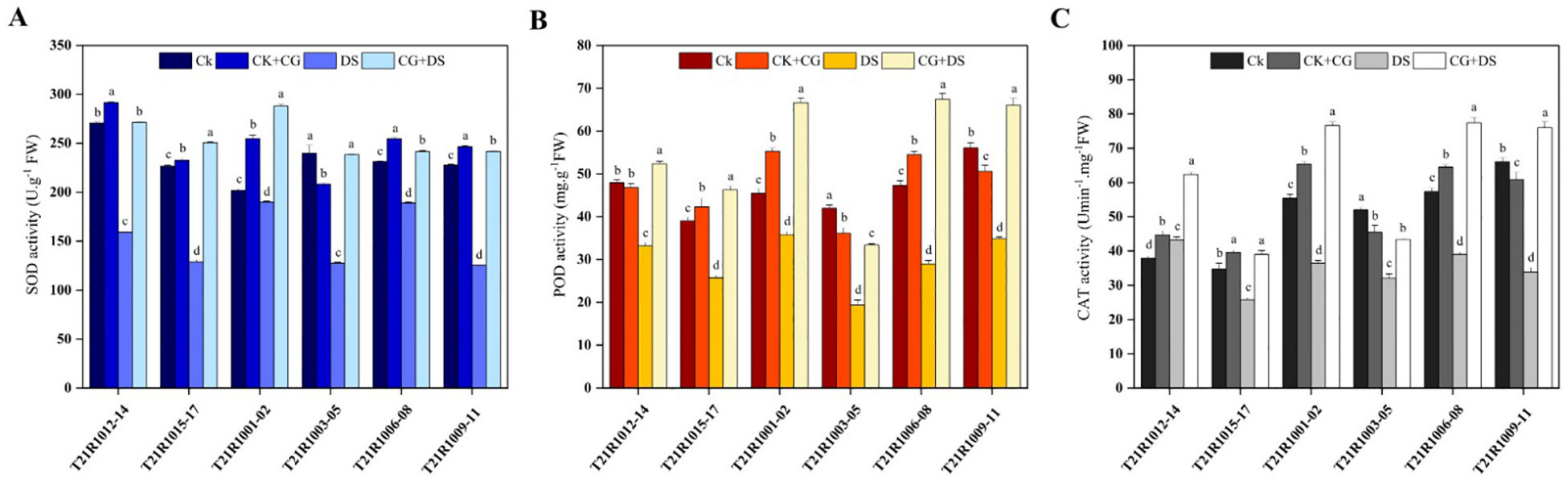
Impacts of seed priming using a combination of sodium carboxymethyl cellulose and gum Arabic (10% CG) on the activity levels of **(A)** superoxide dismutase, SOD, **(B)** peroxidase, POD, and **(C)** catalase CAT. Vertical bars above columns indicate the standard deviation (SD). Mean values followed by different letters are significantly (*P*< 0.01) different from each other according to Duncan’s multiple range test. CK, control seeds (seeds pretreated with H_2_O germinated under normal conditions without any imposed stress); CK+CG, seeds pretreated with 10% CG germinated under normal conditions; DS, seeds pretreated with H_2_O germinated under drought stress; CG+DS, seeds pretreated with 10% CG under drought stress; FW, fresh weight.

Furthermore, the activity of SOD, an antioxidant enzyme, significantly changed in response to water stress. Specifically, the drought stress treatment showed a significant increase in SOD activity compared to normal conditions. Additionally, the CG and drought stress treatment exhibited a further increase in SOD activity (**Figure 7A**). This indicates that seed priming with CG can enhance the antioxidant defense system, as reflected by the increased SOD activity under drought stress (**Figure 7**).

Regarding other antioxidant enzymes, water stress was found to inhibit POD activity but improve CAT activity in both the drought stress, and CG and drought stress treatments (**Figures 7B, C**). However, seedlings germinated from seeds primed with CG showed significantly higher POD and CAT activities compared to seedlings without CG priming (**Figures 7B, C**). This suggests that CG priming can enhance the activities of these antioxidant enzymes, potentially contributing to better protection against oxidative damage caused by drought stress.

### 3.5 Effects of seed priming on EC under flooding conditions

**Figure 8:**
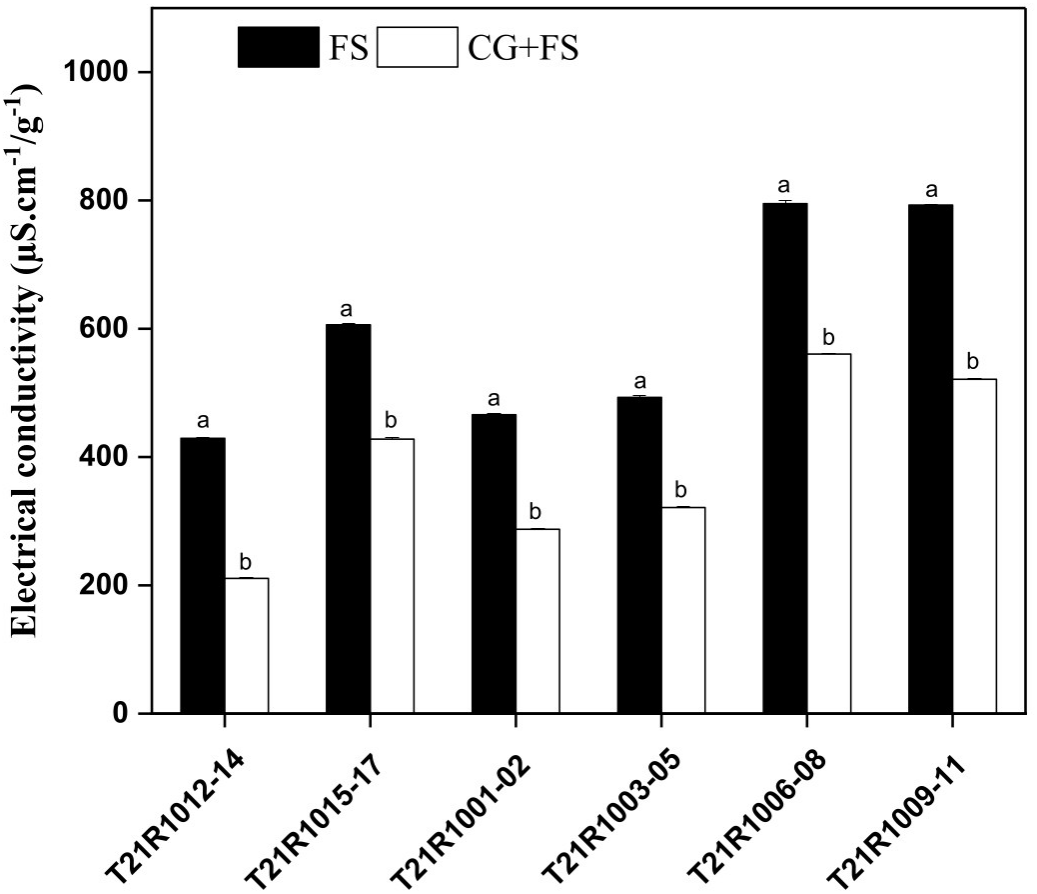
Electrical conductivity (EC) of primed and non-primed soybeans seeds of assessed varieties at 72 h under flooding stress. Vertical bars above columns indicate the standard deviation (SD). Mean values followed by different letters are significantly (*P*< 0.01) different from each other according to Duncan’s multiple range test. FS, control seeds under flooding stress; CG+FS, seeds pretreated with 10% CG under flooding stress. CG, sodium carboxymethyl cellulose incorporated with gum Arabic.

To evaluate the effects of the different seed priming treatments, the EC and GR of six different soybean varieties were examined under control and seed flooding stress. The highest EC was observed in soybean T21R1006-08 and T21R1009-11 under seed flooding stress without seed priming treatments (**Figure 8**).

In seed priming treatments, the EC was significantly reduced in the case of all soybean varieties compared to unprimed seeds under seed flooding stress (**Figure 8**). In the case of 10% CG treatment, the T21R1012-14 variety showed significantly lower EC. The other three varieties have higher EC but have no significant variation of EC among them (**Figure 8**).

## 4 Discussion

Soybean cultivation is critically challenged by abiotic stresses, primarily drought and flooding, which are increased by climate change (Staniak et al., 2023). These environmental stressors disrupt soybean development processes, leading to considerable yield losses and reduced crop productivity (Chen et al., 2016; Mohsen et al., 2023). Drought and flooding stresses directly impact GR, seedling vigor, and the overall health of soybean plants (Mohsen et al., 2023). Given that traditional cultivation methods are often inadequate in mitigating the adverse effects of these stressors, there is a pressing need for innovative strategies such as seed priming to enhance plant resilience (Saberi Riseh et al., 2023). Seed priming, a pre-sowing treatment, displays great promise in preparing seeds to tolerate harsh environmental conditions (El-Sanatawy et al., 2021b). By pre-activating certain metabolic pathways, seed priming facilitates synchronized germination and uniform crop establishment, which is crucial for optimal growth in stressful environments (El-Sanatawy et al., 2021b; Mohsen et al., 2023).

Seed priming using a combination of SCMC and GA (10% CG) showed significant potential in mitigating the effects of drought stress. SCMC is a cellulose derivative with hydrophilic properties, allowing it to retain large amounts of water and maintain a moist microenvironment around seeds (Dingley et al., 2024). This property is critical in drought-prone areas, as it ensures the availability of water for seed imbibition and metabolic activation. GA, sourced from *Acacia* tree sap, shares similar hydrophilic and water-retention capabilities. GA biocompatible nature ensures that it does not introduce toxic residues, supporting sustainable agricultural practices (Badwaik et al., 2022). Both SCMC and GA can be modified chemically to specific environmental needs or seed types, offering a customizable approach to seed priming. The distinctive chemical structures of these compounds facilitate incorporation of nutrients, growth boosters, or stress-mitigating agents, rendering them adaptable tools in agricultural stress management (Mohsen et al., 2023; Dingley et al., 2024).

The results of present study confirmed that seed priming using combination of SCMC and GA (10% CG) significantly enhanced drought tolerance in soybean varieties. Primed seeds exhibited higher GR and percentages, improved ShL and RL, and increased fresh and dry weight compared to unprimed seeds. The reduced MDA content in primed seeds under drought stress suggests that 10% CG priming effectively mitigates oxidative damage by stabilizing cellular membranes. The enhancement of antioxidant enzyme activities, including SOD, CAT, and POD, further supports this protective effect. These enzymes play crucial roles in detoxifying ROS, thus preventing cellular damage and ensuring the maintenance of normal metabolic functions under stress conditions (Hasanuzzaman et al., 2020; Desoky et al., 2023; Mansour et al., 2023).

The coordinated action of these antioxidant enzymes helps maintain redox homeostasis and protects plants from oxidative damage (Desoky et al., 2023). Flooding stress is characterized by oxygen deficiency in the root zone, which impairs root respiration and nutrient uptake (Yijun et al., 2022). The present findings indicated that 10% CG seed priming still provided some advantages under flooding conditions, such as improved GR and reduced EC, but the overall efficacy was lower compared to drought stress. This suggests that flooding stress requires more complex adaptations, and seed priming alone may not be sufficient. The limited effectiveness of CG priming under flooding stress highlights the need for additional or alternative strategies, such as developing soybean varieties with enhanced tolerance to anaerobic conditions or employing advanced soil and water management practices.

Several studies highlighted the potential of seed priming to improve soybean resilience to environmental stresses, offering a promising strategy for achieving more stable agricultural yields under current climate fluctuations (El-Sanatawy et al., 2021a). However, research specifically examining the use of SCMC and GA as seed priming agents for enhancing drought and flooding tolerance in soybean remains limited. Nevertheless, Vanitha et al. (2024) explored the effects of seed priming with silicon dioxide nanoparticles (SiO_2_ NPs) on soybean under drought stress. While this study focused on SiO_2_ NPs, it highlighted the broader context of using various priming agents to enhance drought tolerance in soybean (Vanitha et al., 2024). The findings revealed that seeds treated with SiO_2_ NPs exhibited improved GR and seedling vigor under drought conditions, suggesting that seed priming with appropriate agents can be an effective strategy to mitigate the adverse effects of drought stress on soybean (Vanitha et al., 2024).

Additionally, Tamindžić et al. (2024) explored the impact of simultaneous nutrient priming and biopriming on soybean seed quality and health. The current study focused on combining nutrient priming with biopriming; it highlights the potential of seed priming techniques, including those involving substances like SCMC and GA to improve soybean performance under stress conditions. On the other hand, Ali et al. (2021b); Gong et al. (2023); Ali et al. (2024); Gan et al. (2024) documented that SCMC and GA reduced ROS levels, minimize electrolyte leakage (EL), and preserve membrane integrity, all of which are crucial for sustaining plant health under stress. Hence, the treatments of SCMC and GA enhance the AsA-GSH cycle and facilitate ROS detoxification, significantly boosting plant tolerance to oxidative stress (Fatma et al., 2016; Ali et al., 2021a; Naqve et al., 2021; Yu et al., 2022; Zhang et al., 2024).

By activating the antioxidant defence system, seed priming maintains the structural and functional integrity of chloroplasts, ensuring efficient energy production and reducing photodamage under drought and flooding stresses (Khan et al., 2022; Houmani et al., 2024; Yadav et al., 2024).

## 5 Conclusion

SCMC and GA represent a natural, eco-friendly, and easily prepared strategy that offers multiple efficient anti-stress mechanisms to support plant growth and productivity under stress conditions. Water limitation significantly impairs soybean germination and morphological traits, reducing SFW and SDW while increasing oxidative stress markers and membrane lipid peroxidation. However, these negative effects can be mitigated by using a seed-priming approach with SCMC and gum Arabic (10% GC). This strategy effectively enhanced soybean germination and seedling vigor under drought stress by reducing damage through the activation of both non-enzymatic and enzymatic antioxidant defenses and minimizing membrane lipid peroxidation. Seed priming using 10% CG enhanced drought tolerance by modulating multiple physiological and biochemical pathways, contributing to the development of resilient varieties for sustainable agriculture. Furthermore, the study demonstrated that while 10% CG seed priming offers some benefits under flooding stress, such as improved germination and reduced electrolyte conductivity, its overall efficacy remains limited compared to drought stress. This underscores the necessity for additional strategies, including breeding flood-tolerant soybean varieties and implementing advanced soil and water management practices.

## Data Availability

The raw data supporting the conclusions of this article will be made available by the authors, without undue reservation.
